# Efficacy of brain–computer interface with functional electrical stimulation, transcranial direct current stimulation, and conventional therapy on upper limb recovery after stroke: a systematic review and network meta-analysis

**DOI:** 10.3389/fneur.2025.1643536

**Published:** 2025-11-13

**Authors:** Li Zhang, Meng Zhang, Yi Zhang, Na Li, Jihui Hu, Xiapei Peng

**Affiliations:** Department of Neurology, The Central Hospital of Wuhan, Tongji Medical College, Huazhong University of Science and Technology, Wuhan, China

**Keywords:** stroke, upper limb function, brain-computer interface, functional electrical stimulation, transcranial direct current stimulation

## Abstract

**Objective:**

To systematically evaluate and rank the efficacy of brain-computer interface-based functional electrical stimulation (BCI-FES), transcranial direct current stimulation (tDCS), functional electrical stimulation (FES), conventional therapy (CT), and their combination (BCI-FES + tDCS) on upper limb functional recovery after stroke, and to compare the advantages of different intervention combinations through network meta-analysis, providing evidence-based medicine for clinical practice.

**Methods:**

A network meta-analysis method was used to comprehensively compare the efficacy of BCI-FES, tDCS and conventional motor rehabilitation in upper limb rehabilitation of stroke survivors. Statistical analysis was performed using R and Stata software, including direct meta-analysis and network meta-analysis. The direct meta-analysis used mean difference (MD) and its 95% confidence interval (CI) as effect size indicators. The network meta-analysis was performed within a Bayesian framework using the gemtc package in R.

**Results:**

A total of 13 relevant studies were finally included, comprising 11 two-arm studies and 2 three-arm studies, with a total of 777 subjects. Direct comparison meta-analysis showed: BCI-FES vs. CT MD = 6.01 (95%CI: 2.19, 9.83); BCI-FES vs. FES MD = 3.85 (95%CI: 2.17, 5.53); BCI-FES vs. tDCS MD = 6.53 (95%CI: 5.57, 7.48); BCI-FES + tDCS vs. BCI-FES MD = 3.25 (95%CI: −1.05, 7.55); BCI-FES + tDCS vs. tDCS MD = 6.05 (95%CI: −2.72, 14.82). BCI-FES showed significantly better effects than CT, FES and tDCS in improving FMA. Network meta-analysis: The inconsistency model was not significant (*p* = 0.060), so the consistency model was adopted. The efficacy ranking was BCI-FES + tDCS (98.9), BCI-FES (73.4), tDCS (33.3), FES (32.4), CT (12.0). BCI-FES and BCI-FES + tDCS were significantly better than CT, but there was no statistically significant difference compared with FES and tDCS.

**Conclusion:**

The combined application of BCI-FES and tDCS appears promising for upper limb rehabilitation after stroke, with potential therapeutic advantages arising from multimodal promotion of neuroplasticity. However, given the small number of trials, methodological variability, and risk of bias, this conclusion should be considered exploratory and hypothesis-generating rather than definitive guidance. Future studies should further verify its clinical benefits through standardized stimulation protocols, individualized parameter optimization and multicenter long-term follow-up studies, to promote the translational application of brain-computer interface technology in the field of neurorehabilitation.

**Systematic review registration:**

INPLASY202550066.

## 1 Introduction

Stroke, the second leading global cause of death and a primary contributor to long-term disability, results in approximately 13 million new cases annually, with more than 80% of survivors experiencing persistent upper limb motor impairment (1). According to the World Health Organization’s disability weight metrics, post-stroke upper limb dysfunction carries a severity coefficient of 0.35—substantially higher than the 0.24 assigned to lower limb deficits—highlighting its profound impact on functional independence (2). Upper limb impairments extend beyond weakness and incoordination, reflecting widespread disruption of sensorimotor integration and cortical reorganization. While restoring upper extremity function remains a cornerstone of stroke rehabilitation, conventional approaches such as constraint-induced movement therapy and task-specific training exhibit limited efficacy in severe cases (3). Traditional task-oriented rehabilitation yields only modest gains (e.g., 6–8 points on the FMA-UE). With nearly two-thirds of patients experiencing significant functional limitations 6 months post-stroke, the development of more advanced interventions is clearly warranted (4).

Brain-computer interface (BCI) technology, which decodes cortical activity to generate device control signals in real time, has emerged as a transformative approach for motor recovery. By closing the sensorimotor loop through neurofeedback, BCI systems promote adaptive neuroplasticity and functional restoration (5, 6). Recent advances integrating BCI with functional electrical stimulation (FES)—termed BCI-FES—have demonstrated particular promise (7). FES directly activates paralyzed muscles via precisely timed electrical pulses, simultaneously reinforcing peripheral neuromuscular pathways and facilitating central circuit reorganization (8). When synchronized with EEG-detected movement intention in a BCI-FES paradigm, this closed-loop system enhances proprioceptive feedback and strengthens efferent-reafferent coupling, driving superior functional gains compared to open-loop stimulation (9–11). Complementary noninvasive neuromodulation techniques such as transcranial direct current stimulation (tDCS) have been investigated for stroke rehabilitation. Anodal tDCS can transiently enhance cortical excitability and potentially facilitate motor recovery; however, the literature remains conflicting. Several randomized trials and meta-analyses report minimal or inconsistent clinical benefits in post-stroke upper limb function, raising concerns about its standalone efficacy (12, 13). Importantly, tDCS has not been approved by the U.S. Food and Drug Administration (FDA) for stroke rehabilitation, which is a critical translational consideration. Moreover, recent randomized controlled trials have also reported negative or null findings, further suggesting that the clinical value of tDCS alone remains uncertain (12, 13). Thus, while tDCS may augment other interventions such as BCI-FES, its role as an independent therapy remains uncertain and requires cautious interpretation within this review.

Despite accumulating evidence supporting the clinical potential of BCI-based therapies, critical knowledge gaps remain concerning their comparative effectiveness and optimal integration. Several systematic reviews and meta-analyses have established the efficacy of BCI-FES for upper limb recovery compared to conventional therapy (6, 7). Similarly, numerous studies and Cochrane reviews have investigated tDCS, although its standalone benefits remain a subject of debate due to inconsistent findings across trials (12, 13). Furthermore, conventional FES has long been a staple in post-stroke rehabilitation, with documented benefits for muscle activation and motor control (8, 14).

However, the existing literature is characterized by fragmented comparisons. While pairwise meta-analyses have typically compared BCI-FES to conventional care or FES to sham, they fail to address a more pressing clinical question: Among this new generation of neurotechnologies and established modalities, which intervention, or combination thereof, yields the superior therapeutic effect? For instance, it remains unclear whether the closed-loop, intention-driven paradigm of BCI-FES offers a significant advantage over the non-invasive cortical modulation of tDCS, or how their combination might synergize. To date, no systematic synthesis has directly evaluated the efficacy hierarchy among BCI-coupled FES, standalone FES, tDCS, and their combined protocols. This lack of a comprehensive comparative framework limits evidence-based clinical decision-making.

This network meta-analysis (NMA) therefore provides the first comprehensive and simultaneous comparison of these interventions. By integrating both direct and indirect evidence from the available randomized controlled trials, this study aims to: (1) determine the relative efficacy of BCI-FES, tDCS, FES, conventional therapy, and the combined approach (BCI-FES + tDCS); (2) rank these interventions according to their probability of being the most effective; and (3) establish an evidence-based hierarchy to inform the development of precision rehabilitation frameworks tailored to individual patient profiles.

## 2 Methods

### 2.1 Study design

This study utilized a NMA to compare the therapeutic efficacy of BCI-FES, tDCS, and conventional motor rehabilitation for upper limb functional recovery in stroke survivors.

This systematic review and network meta-analysis was registered with the International Platform of Registered Systematic Review and Meta-analysis Protocols (INPLASY), 202,550,066.

### 2.2 Literature search strategy

A comprehensive search strategy was implemented across seven major electronic databases: PubMed (MEDLINE), Embase, Cochrane Central Register of Controlled Trials, Web of Science Core Collection, along with three prominent Chinese databases (CNKI, Wanfang, and VIP). The search encompassed all available records from each database’s inception through April 2025.

Our search algorithm incorporated:

- Controlled vocabulary terms (MeSH in PubMed/MEDLINE, Emtree in Embase).
- Free-text keywords including but not limited to:

- Neurotechnology-based interventions (“BCI,” “brain-machine interface”);
- Neuromodulation techniques (“tDCS,” “non-invasive brain stimulation”);
- Rehabilitation modalities (“FES,” “electromyography-triggered stimulation”);
- Clinical populations (“cerebrovascular accident,” “hemiparesis”);
- Functional outcomes (“motor recovery,” “upper extremity function”).

To ensure comprehensive literature coverage, backward citation tracking of all eligible articles was performed, and relevant systematic reviews were examined. This supplementary hand-searching strategy was implemented to identify any potentially relevant studies not retrieved through the primary electronic search.

### 2.3 Study selection

A standardized three-stage screening process (initial screening → full-text assessment → data compliance verification) was implemented by two independent researchers. The study inclusion and exclusion criteria were defined *a priori* according to the PICOS framework (Participants, Interventions, Comparators, Outcomes, Study design) as follows:

- P (Participants): Adult patients (age ≥ 18 years) diagnosed with ischemic or hemorrhagic stroke, at a chronic or subacute stage (disease duration ≥1 month), who exhibited upper limb motor dysfunction (Brunnstrom stage ≥ II). Studies involving patients with non-stroke etiologies (e.g., brain trauma, spinal cord injury), severe cognitive impairment (MMSE <18), or complete upper limb paralysis were excluded.
- I (Interventions): The experimental intervention must include brain-computer interface-based functional electrical stimulation (BCI-FES). For studies investigating combined interventions, the BCI-FES component had to be clearly described, including trigger thresholds.
- C (Comparators): Eligible comparators included: (1) Active controls: other active interventions (e.g., tDCS, FES alone); (2) Passive controls: conventional motor rehabilitation (physical therapy/occupational therapy) or sham stimulation (e.g., sham tDCS).
- (Outcomes): The primary outcome of interest was the upper limb motor score of the Fugl-Meyer Assessment (FMA-UE). Studies that did not report FMA-UE scores or from which numerical data (mean, standard deviation) could not be extracted were excluded.
- S (Study design): Only randomized controlled trials (RCTs) were included. Non-randomized studies, cohort studies, case series, conference abstracts, and studies with incomplete outcome data were excluded.

Additionally, studies were excluded for the following reasons: (1) mixed interventions without separable upper limb outcome reporting; (2) duplicate publications or overlapping patient cohorts; (3) non-English or non-Chinese full texts being unavailable; and (4) substandard intervention parameters (e.g., tDCS current intensity <1 mA).

The inclusion and exclusion criteria based on PICOS principles are presented in Table 1.

**Table 1:** Literature inclusion and exclusion criteria.

Category	Specific criteria	Detailed explanations
P (Population)	Adult patients with ischemic/hemorrhagic stroke (disease duration ≥1 month)	Exclusions
	Upper limb motor dysfunction (Brunnstrom stage ≥II)	Non-stroke etiologies (e.g., brain trauma, spinal cord injury)
	Age ≥18 years	Severe cognitive impairment (MMSE <18)
		Complete upper limb paralysis
I (Intervention)	Must include BCI-FES	For combined interventions
		BCI-FES must specify trigger thresholds
		Composite interventions (e.g., tDCS + rehab) should be categorized separately
C (Comparator)	Active control: Other interventions (e.g., BCI vs. tDCS)	Examples
	Passive control: Conventional rehab (PT/OT) or sham stimulation (e.g., tDCS placebo)	BCI group vs. conventional rehab group
		FES group vs. sham stimulation group
O (Outcomes)	Upper limb Fugl-Meyer Assessment (FMA) score	Exclude: Studies without extractable data (e.g., figures/tables lacking numerical values)
S (Study design)	Included: Randomized controlled trials (RCTs)	Data requirements
	Excluded: Non-randomized studies (cohort/case series), conference abstracts, incomplete data	Baseline data reporting
		Outcome metrics as mean ± SD or convertible formats
Additional exclusion criteria	Mixed interventions without separate upper limb outcome reporting	
	Duplicate publications or phased data from the same study	
	Non-English/Chinese literature with unavailable full texts	
	Substandard intervention parameters (e.g., tDCS current <1 mA)	
Screening key points	Initial screening excludes non-stroke/non-upper limb interventions (e.g., gait training)	
	Full-text review for parameter compliance and data completeness	
	Preset subgroup analysis by disease duration (≤6 months vs. >6 months)	

### 2.4 Data extraction and quality assessment

Two researchers independently conducted study selection, data extraction, and quality assessment. For study selection, initial screening was performed based on titles and abstracts to exclude clearly ineligible studies, followed by full-text review for final inclusion determination. Extracted data included: basic study characteristics (e.g., authors, publication year), participant characteristics (e.g., sample size, age, stroke type), intervention details (e.g., stimulation parameters, training frequency and duration), and outcome measure data. The methodological rigor of the included studies was critically appraised using the Cochrane Collaboration’s tool for assessing risk of bias. This comprehensive evaluation encompassed six key domains: randomization procedures, allocation concealment methods, blinding implementation, handling of incomplete outcome data, potential selective reporting, and other sources of bias. Any disagreements among reviewers regarding quality assessment were systematically addressed through iterative discussion or, when necessary, arbitration by an independent third investigator.

In accordance with the International Classification of Functioning framework, we classified outcomes as impairment-level (FMA-UE) versus activity-level (ARAT, WMFT, and ADL scales), and prespecified FMA-UE as the primary endpoint for pooling, with activity-level measures analyzed as secondary outcomes subject to data availability.

### 2.5 Assessment of the certainty of evidence

The certainty of evidence for each pairwise comparison derived from both the direct meta-analysis and the network meta-analysis was assessed using the Grading of Recommendations, Assessment, Development, and Evaluations (GRADE) framework for network meta-analysis.

The evaluation was conducted by two independent reviewers. The initial certainty of evidence for all randomized controlled trial (RCT) comparisons was set as high, and was then potentially downgraded based on the following five domains: (1) Risk of Bias: Evaluation based on the Cochrane Risk of Bias 2.0 (RoB 2.0) tool. (2) Inconsistency: Assessed by evaluating the heterogeneity (I^2^ statistic) and overlap of confidence intervals in direct estimates, and the statistical inconsistency between direct and indirect evidence in the NMA. (3) Indirectness: Judged based on the relevance of the included populations, interventions, comparators, and outcomes to the review question. (4) Imprecision: Evaluated by examining whether the 95% confidence or credible intervals around the effect estimate crossed the threshold of a minimal clinically important difference (MCID, preset at 5 points for FMA-UE) or included the null effect. (5) Publication Bias: Assessed through funnel plot symmetry, acknowledging the limited power of these tests when the number of studies is small.

### 2.6 Statistical analysis

Statistical analyses were performed using R software (version 4.3.2; R Foundation for Statistical Computing) and Stata software (version 18.0; StataCorp LLC), with each software handling distinct components of the analysis.

For direct meta-analysis, mean difference (MD) with 95% confidence intervals (CI) served as effect size measures. For studies not reporting means and standard deviations, conversion was performed using formulas recommended in the Cochrane Handbook. Heterogeneity was assessed using Higgins I^2^ statistics, with I^2^ ≤ 25% indicating low heterogeneity, 25% < I^2^ ≤ 50% moderate heterogeneity, and I^2^ > 50% substantial heterogeneity. When substantial heterogeneity was present, potential sources were investigated, and random-effects models were applied; otherwise, fixed-effects models were used. Primary meta-analyses were conducted on impairment-level outcomes (FMA-UE), while activity-level outcomes (ARAT, WMFT, ADL) were synthesized as secondary analyses or narratively summarized when pooling was infeasible due to insufficient studies.

For network meta-analysis, a Bayesian framework was employed. The model assumed homogeneity across studies and comparability of indirect comparisons between different interventions. When both direct and indirect comparisons were available, mixed treatment effects models were used, combining results through inverse-variance weighting. Analyses were performed using the gemtc package in R. Inconsistency was evaluated by comparing direct and indirect evidence within the network meta-analysis. If results showed statistical consistency between direct and indirect evidence, consistency models were applied. The surface under the cumulative ranking curve (SUCRA) was used to rank intervention efficacy, with relative effect sizes and 95% credible intervals calculated to provide intuitive references for clinical decision-making.

The network geometry diagram (Figure 1) and the SUCRA ranking plot (Figure 2) were generated using Stata software for efficient and high-quality graphical presentation. The funnel plot (Figure 3) was also generated in Stata. All forest plots for direct comparisons (Figures 4–8) were produced in Stata.

**Figure 1:**
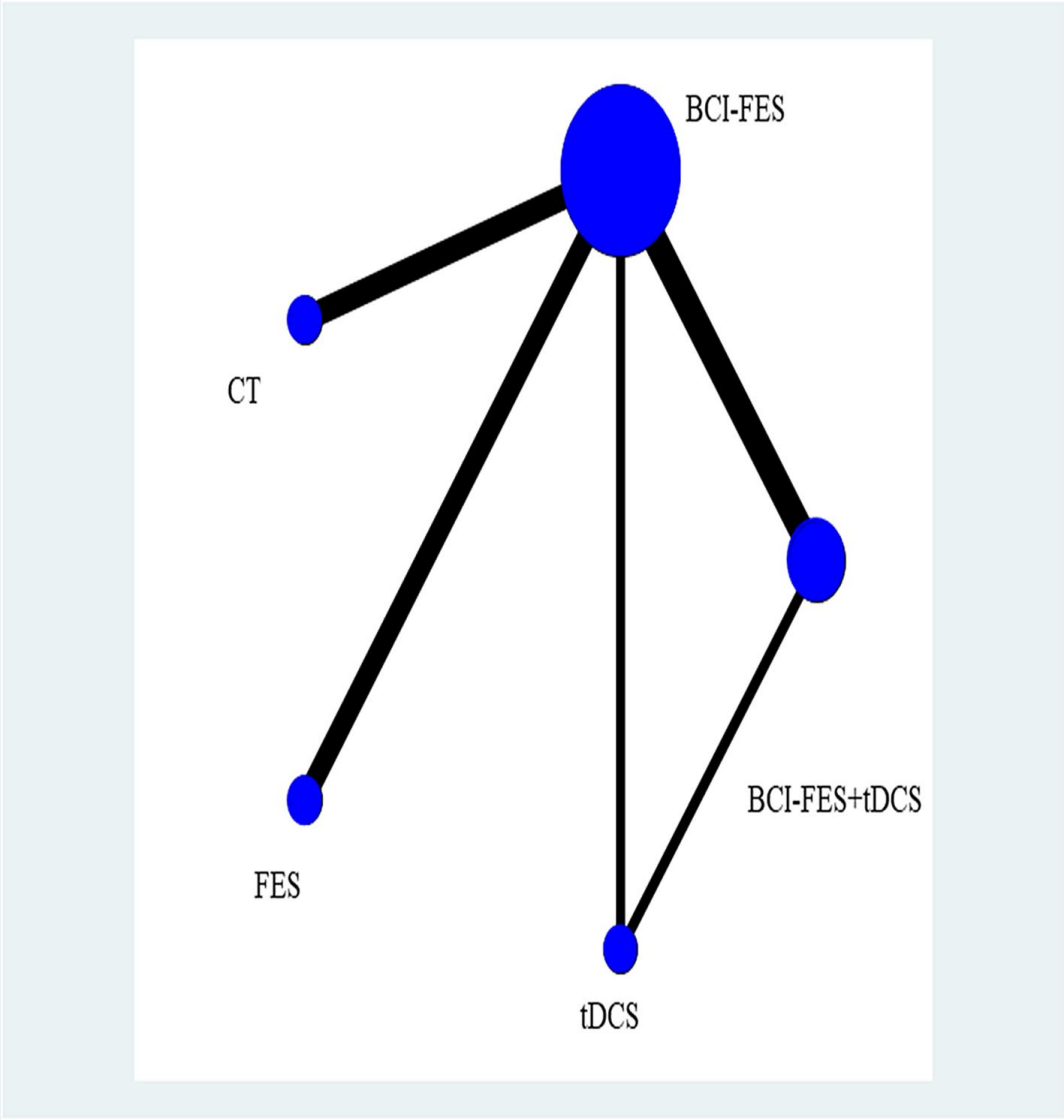
Network relationship diagram.

**Figure 2:**
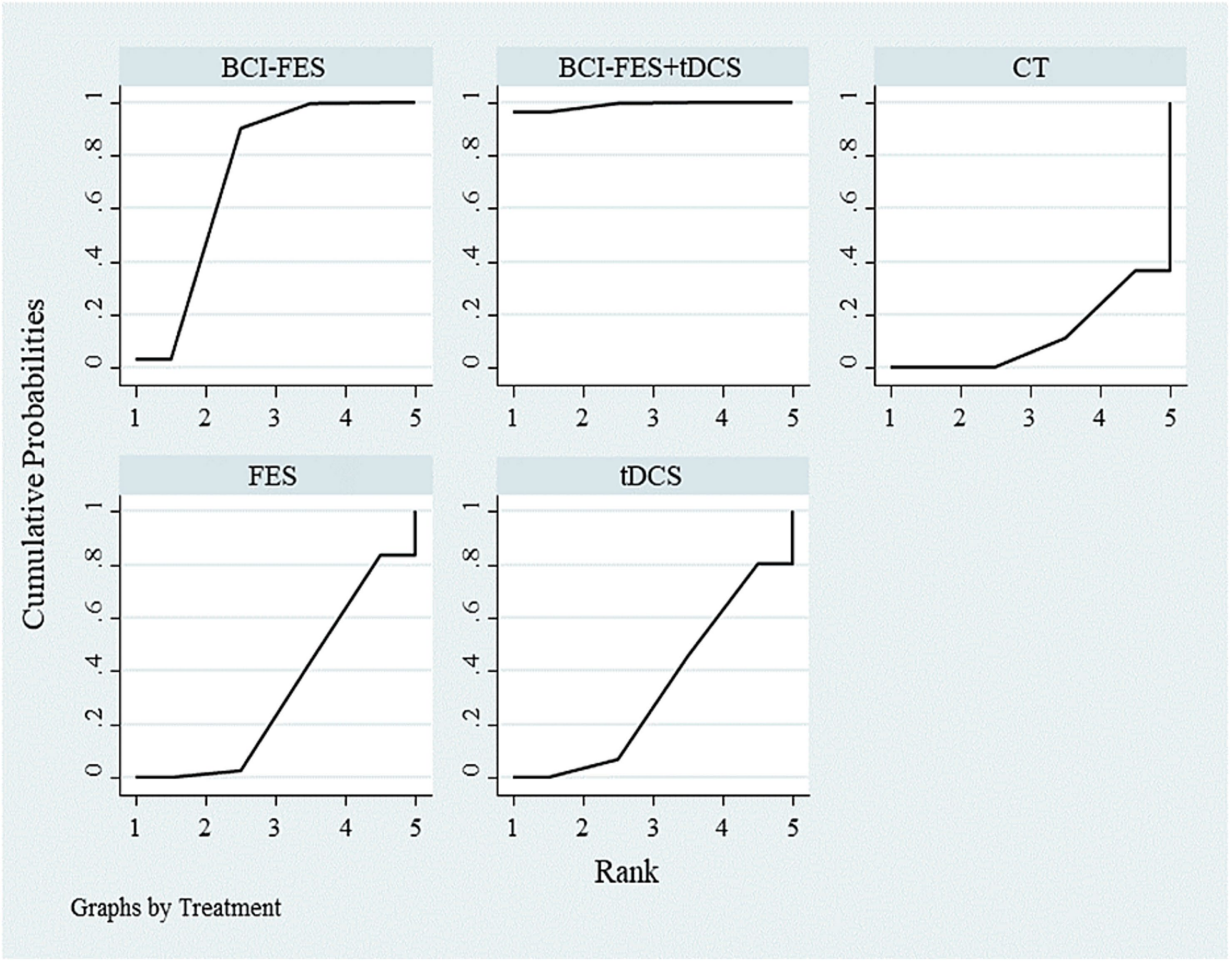
SUCRA ranking of different interventions.

**Figure 3:**
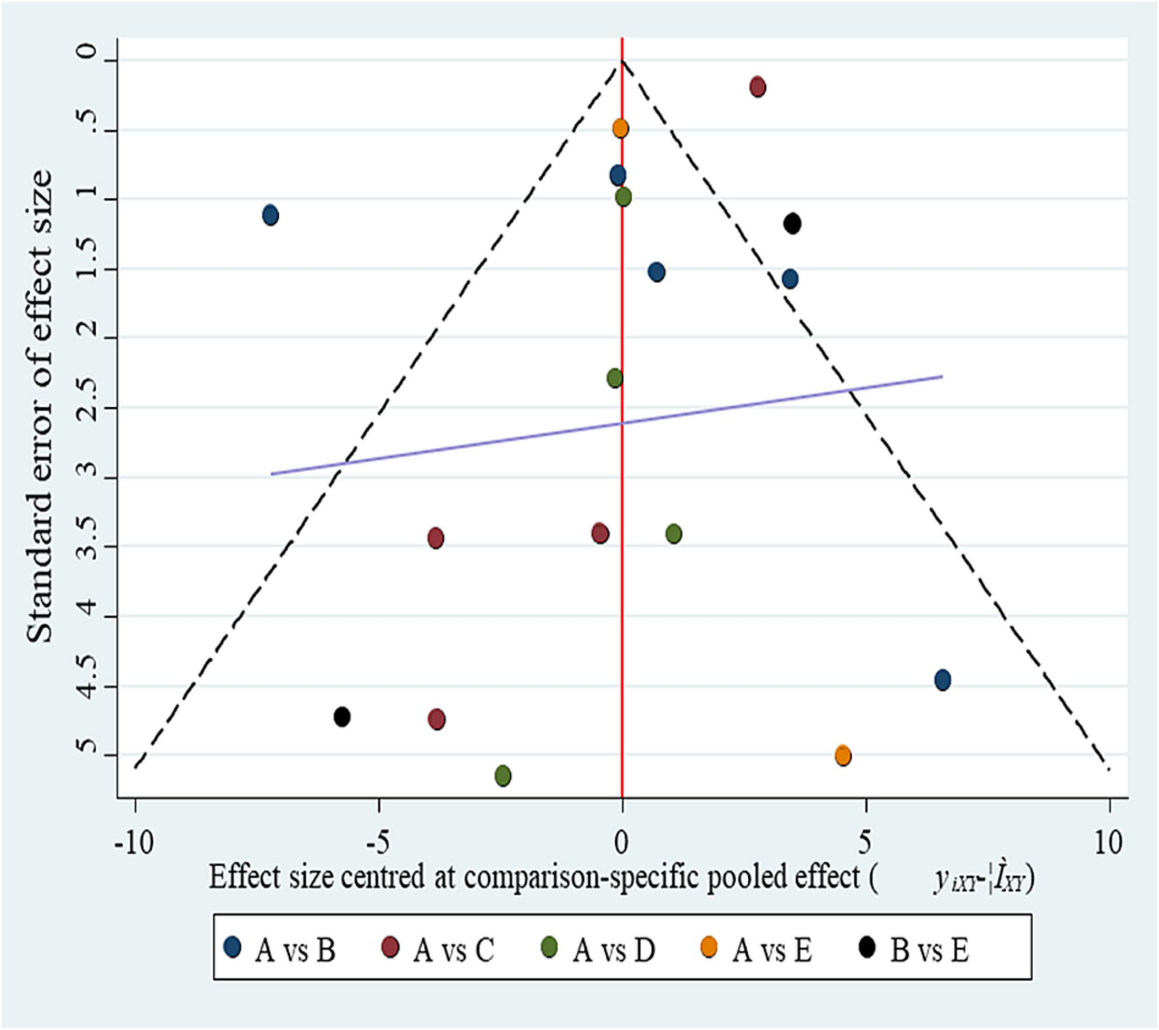
Funnel plot for publication bias. (A) BCI-FES; (B) BCI-FES + tDCS; (C) CT; (D) FES; (E) tDCS. Although the plot appears symmetrical, the small number of studies per comparison (2–4) severely limits the ability to detect small-study effects.

**Figure 4:**
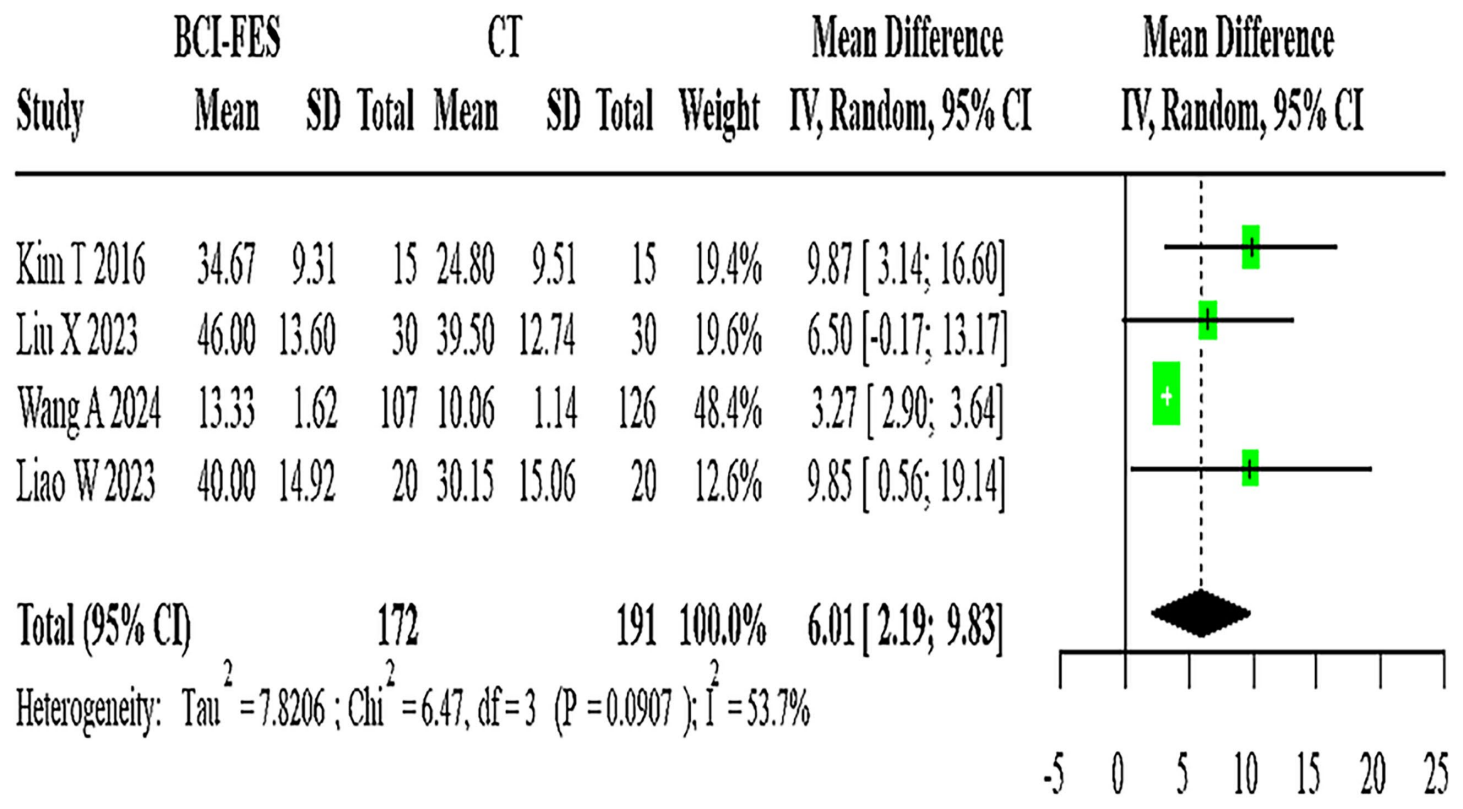
Forest Plot of BCI-FES vs. CT comparison. The pooled MD (6.01, 95% CI: 2.19–9.83) approximates the minimal clinically important difference (MCID ≈ 5–7 points for FMA-UE), suggesting potential clinical relevance.

**Figure 5:**
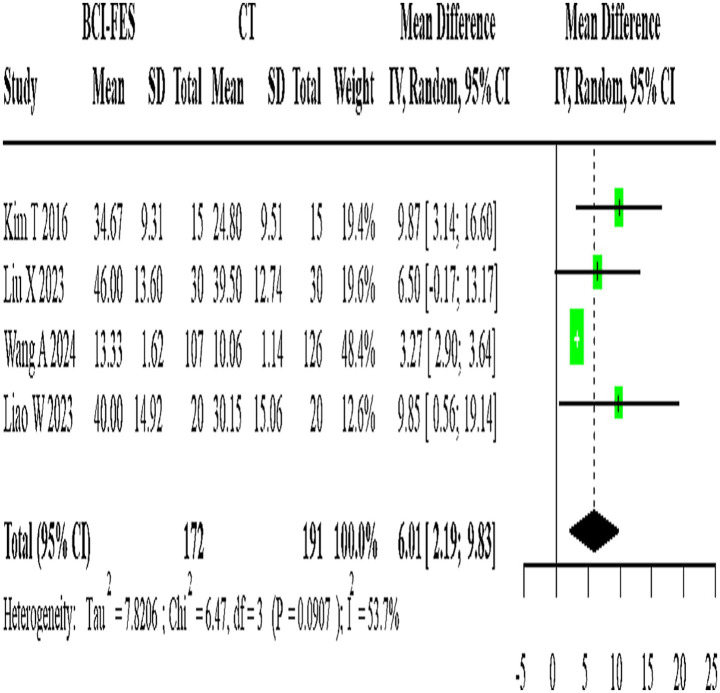
Forest plot of BCI-FES vs. FES comparison.

**Figure 6:**
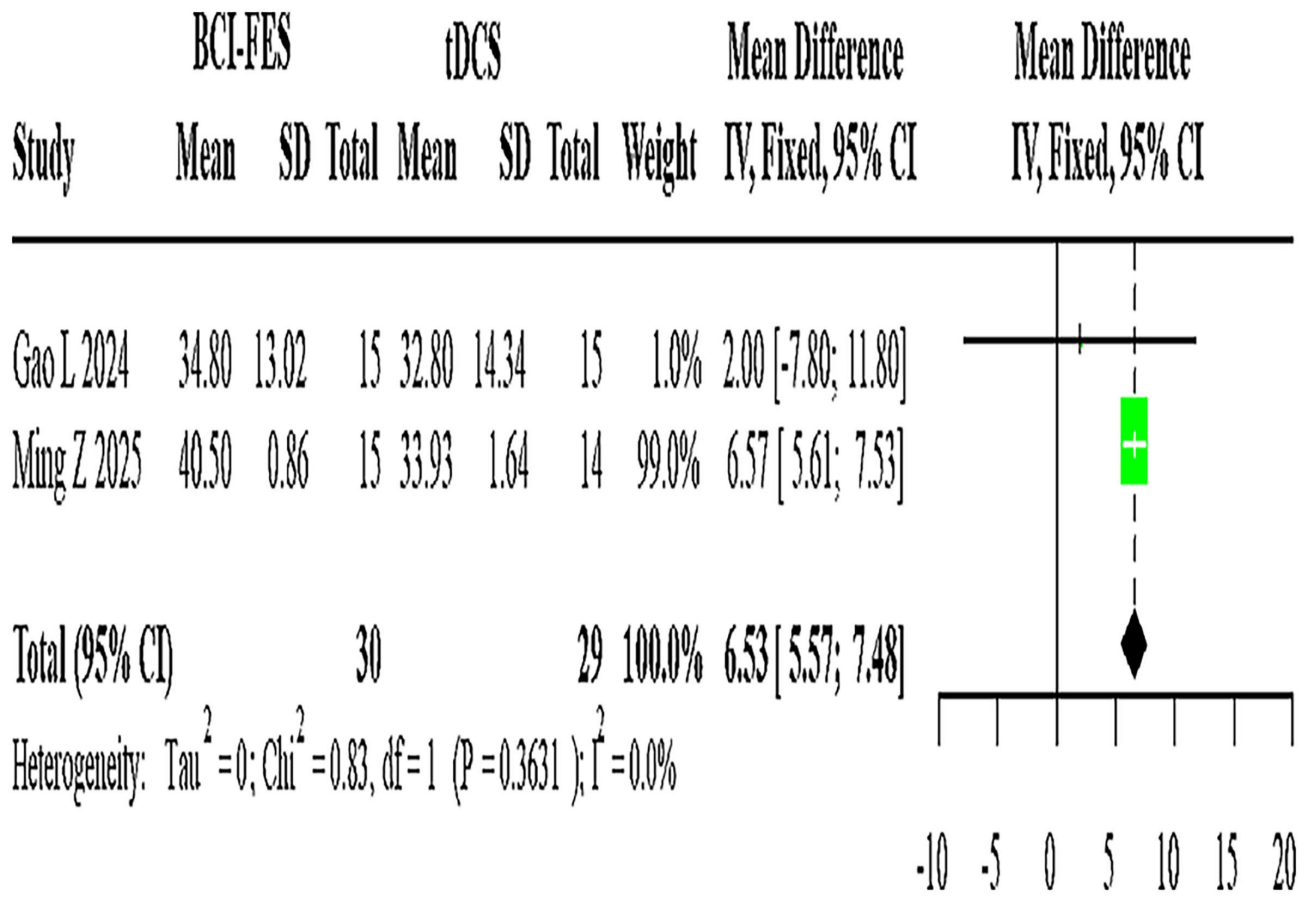
Forest plot of BCI-FES vs. tDCS comparison.

**Figure 7:**
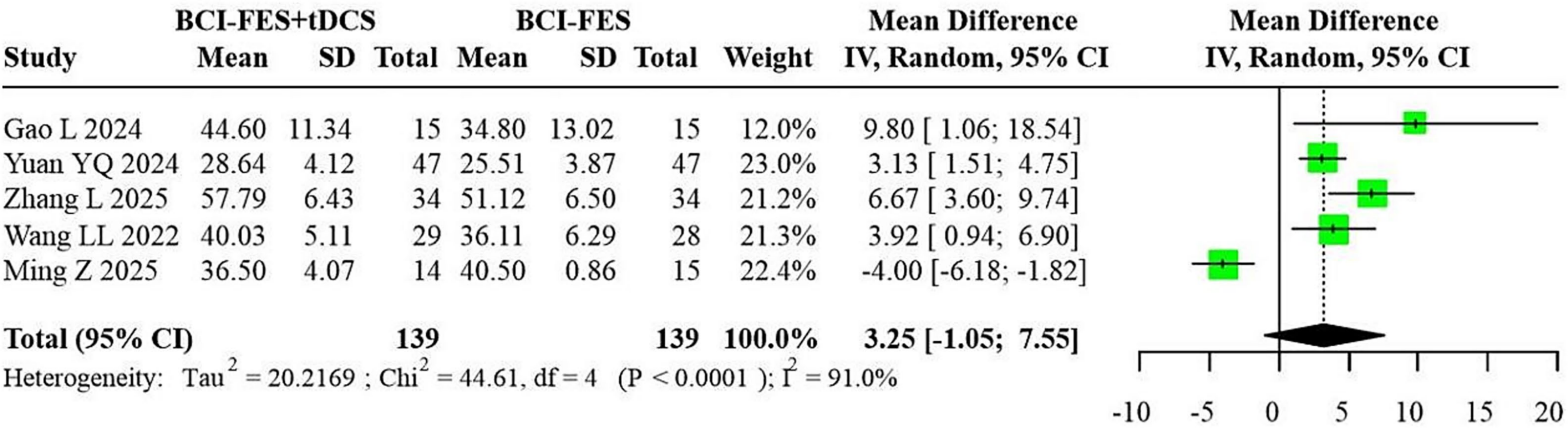
Forest plot of BCI-FES + tDCS vs. BCI-FES comparison.

**Figure 8:**
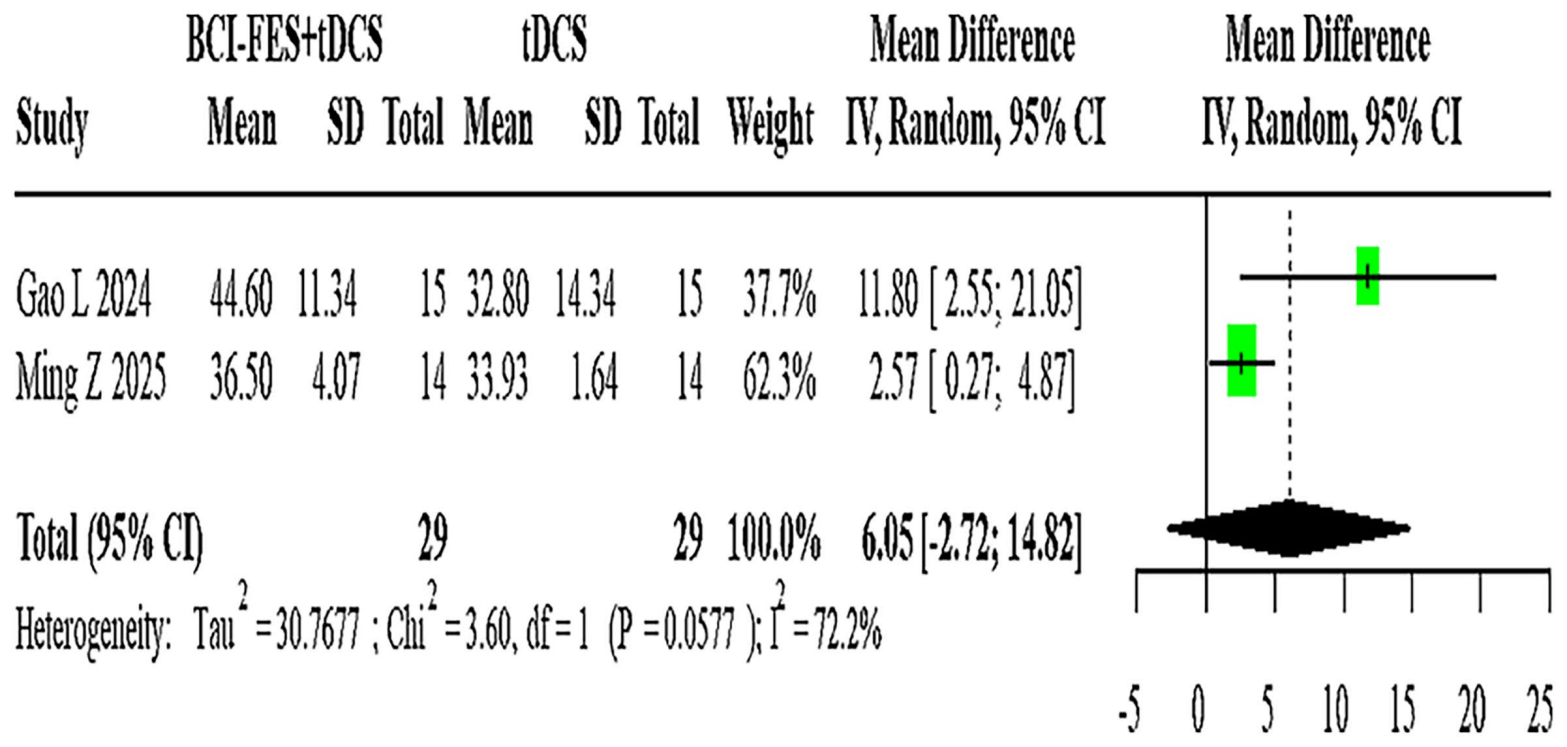
Forest plot of BCI-FES + tDCS vs. tDCS comparison.

## 3 Results

### 3.1 Literature screening process

**Figure 9:**
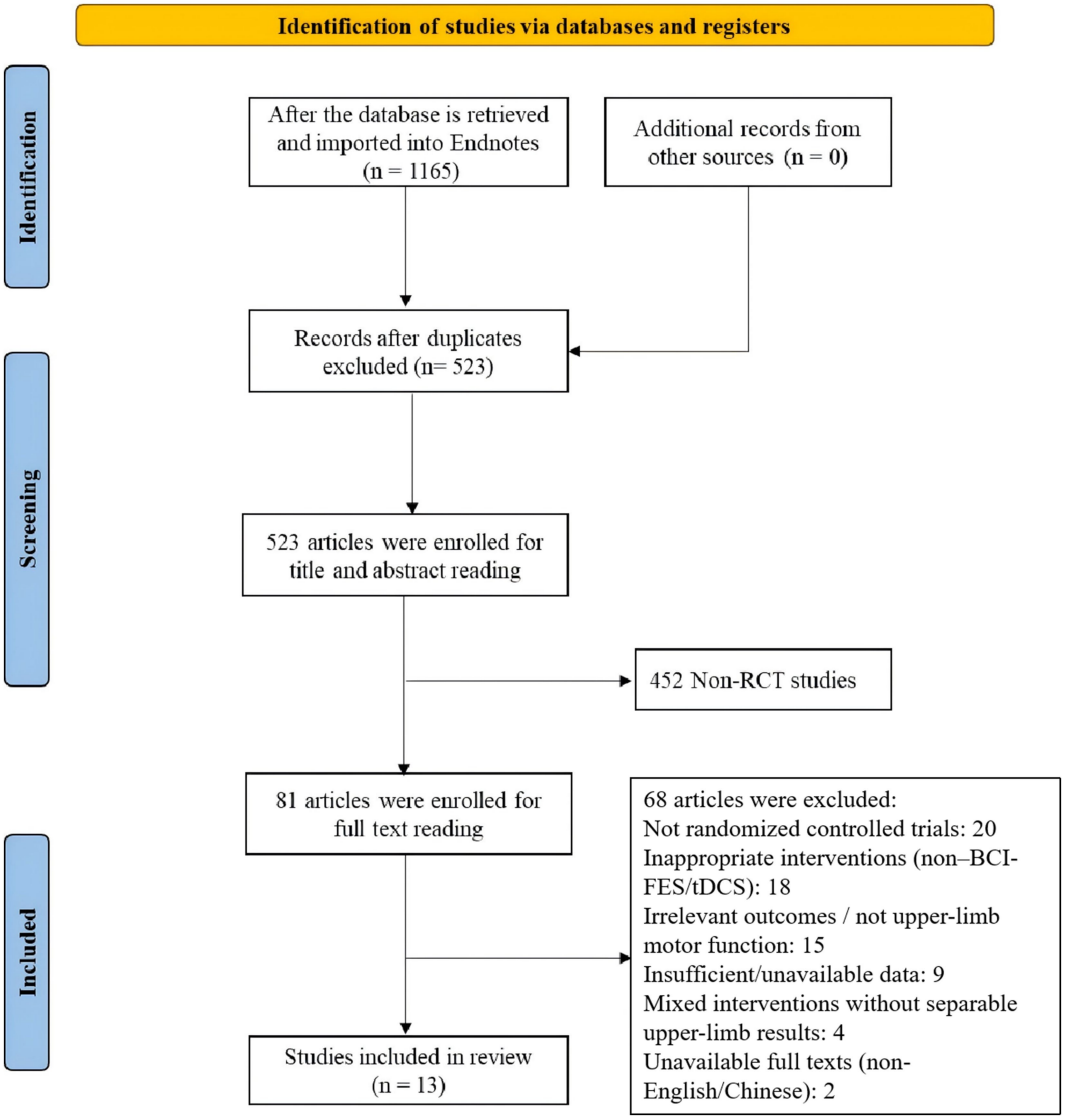
PRISMA flow diagram of study selection.

The PRISMA flowchart was utilized to identify and screen relevant studies. Our search targeted studies investigating the application of BCI-FES, FES, tDCS, and conventional motor rehabilitation in upper limb recovery among stroke survivors. Following a standardized three-stage screening process (initial screening, full-text evaluation, and data compliance verification), 13 studies were ultimately included. The study selection flowchart is presented in Figure 9.

### 3.2 Study characteristics

Among the 13 included studies, 11 employed a two-arm design while 2 utilized a three-arm design, encompassing a total of 777 participants. Interventions assessed included conventional therapy (CT), FES, tDCS, BCI-FES, and BCI-FES-tDCS. After converting three-arm studies into two-arm comparisons, five treatment combinations were analyzed: BCI-FES vs. BCI-FES-tDCS, BCI-FES vs. CT, BCI-FES vs. FES, BCI-FES vs. tDCS, and BCI-FES-tDCS vs. tDCS. Key study characteristics are summarized in Table 2. However, details on intervention parameters—including training dose, frequency, session duration, and stimulation settings (e.g., tDCS current intensity, FES pulse parameters, BCI classification accuracy)—were inconsistently reported across trials. In fact, 7 out of the 13 included studies did not fully report one or more of these critical parameters (Table 2). This highlights a pervasive lack of standardized reporting, which may limit reproducibility and complicate interpretation of between-study differences. To enhance clarity, a dedicated summary table of intervention parameters (Table 2) is provided, while also emphasizing the urgent need for future trials to adopt uniform reporting standards.

**Table 2:** Key study characteristics and intervention parameters.

Author (Year)	Intervention	Control	Course	Sample size	BCI parameters	FES parameters	tDCS parameters	Session duration and frequency
Kim T 2016 (24)	BCI-FES	CT	4 weeks	15 vs. 15	Not reported	Pulse width 300 μs, 35 Hz, intensity NR	N/A	30 min, 5×/week
Lee SH 2022 (25)	BCI-FES	FES	4 weeks	13 vs. 13	Accuracy NR, AO training combined	250 μs, 40 Hz, intensity individualized	N/A	30 min, 5×/week
Liu X 2023 (26)	BCI-FES	CT	3 weeks	30 vs. 30	Motor imagery, accuracy NR	NR	N/A	30 min, 5×/week
Wang A 2024 (27)	BCI-FES	CT	4 weeks	107 vs. 126	Visual feedback, accuracy ~70%	NR	N/A	30 min, 5×/week
Biasiucci A 2018 (7)	BCI-FES	CT	5 weeks	14 vs. 13	EEG-triggered, accuracy NR	NR	N/A	30–45 min, 5×/week
Jang YY 2016 (28)	BCI-FES	FES	6 weeks	10 vs. 10	Motor imagery, accuracy NR	250–300 μs, 30 Hz	N/A	30 min, 5×/week
Tang QM 2021 (29)	BCI-FES	FES	4 weeks	17 vs. 17	Accuracy NR	NR	N/A	30 min, 5×/wk
Gao L 2024 (30)	BCI-FES, BCI-FES + tDCS	tDCS	4 weeks	15 vs. 15 vs. 15	Visual/auditory/motor feedback, accuracy ~70%	NR	2 mA, 20 min, anodal over M1	30 min, 5×/week
Yuan YQ 2024 (31)	BCI-FES + tDCS	BCI-FES	4 weeks	47 vs. 47	Feedback NR	300 μs, 30 Hz	2 mA, 20 min, anodal ipsilesional M1	30 min, 5×/week
Zhang L 2025 (32)	BCI-FES + tDCS	BCI-FES	4 weeks	34 vs. 34	NR	NR	2 mA, 20 min	30 min, 5×/week
Wang LL 2022 (33)	BCI-FES + tDCS	BCI-FES	4 weeks	29 vs. 28	NR	NR	1–2 mA, 20 min	30 min, 5×/week
Liao W 2023 (34)	BCI-FES	CT	3 weeks	20 vs. 20	Motor imagery, accuracy NR	NR	N/A	30 min, 5×/week
Ming Z 2025 (35)	BCI-FES, BCI-FES + tDCS	tDCS	4 weeks	15 vs. 14 vs. 14	Kinesthetic MI, accuracy NR	NR	2 mA, 20 min	30 min, 5×/week

BCI, brain-computer interface; FES, functional electrical stimulation; tDCS, transcranial direct current stimulation; CT, conventional therapy; AO, action observation; MI, motor imagery; NR, not reported.

**Figure 10:**
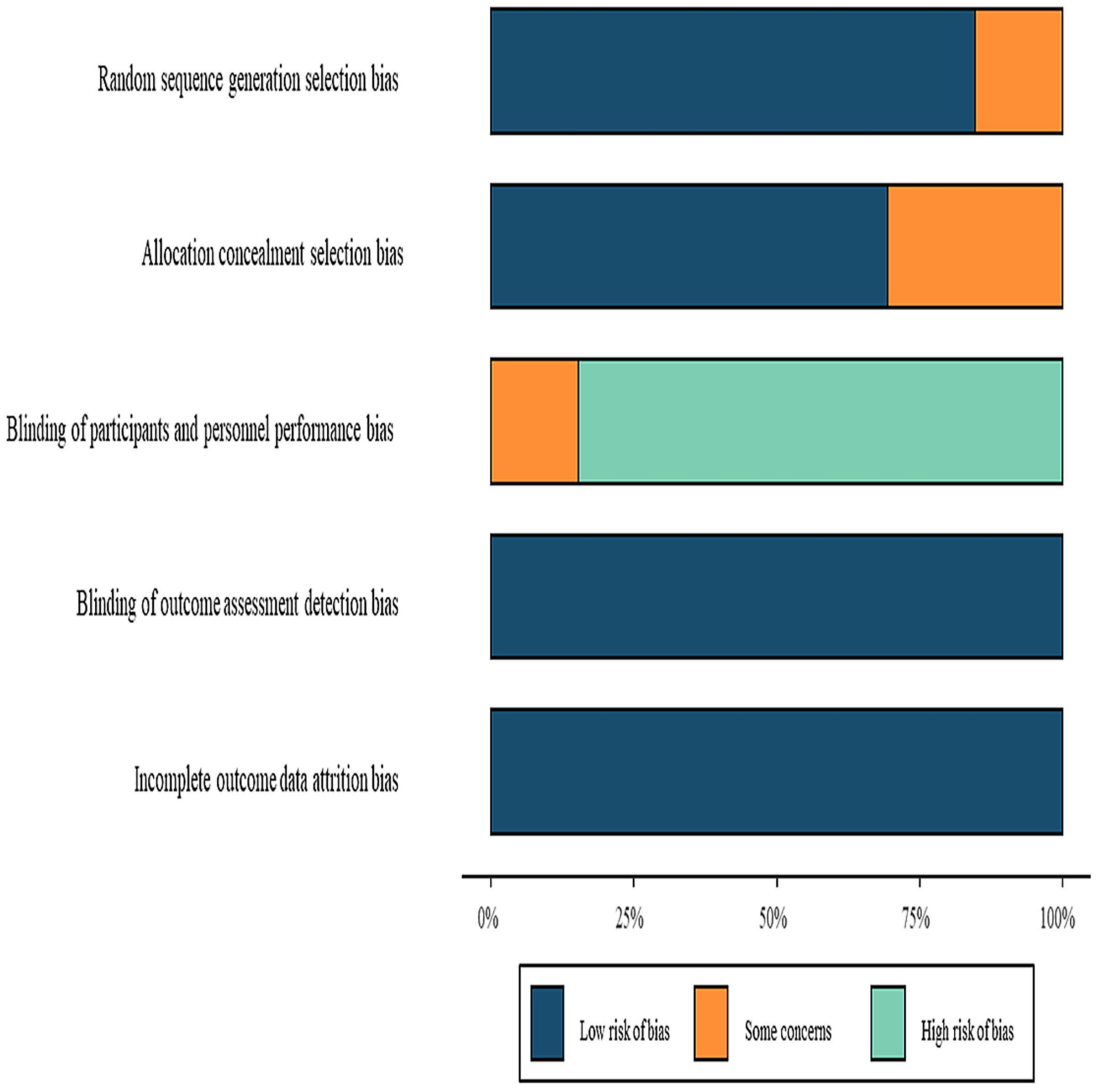
Risk of bias summary.

Quality assessment using ROB 2.0 indicated low risk of bias in missing outcome data and selective reporting. However, concerns were identified regarding randomization procedures, allocation concealment, and outcome measurement. Due to impracticality of blinding participants and intervention providers, most studies focused on blinding outcome assessors, potentially introducing performance bias. Moreover, the majority of included RCTs were small, single-center trials, often lacking pre-registration or detailed protocols. Several trials also presented unclear or high risk of bias in key domains such as randomization, allocation concealment, and blinding. Overall, the methodological quality of the included studies is low to moderate, increasing the risk of selection bias, performance bias, and selective reporting. Therefore, the conclusions of this NMA should be interpreted cautiously. The risk of bias summary is illustrated in Figure 10.

The application of the GRADE framework to the evidence for the primary outcome (FMA-UE) found the certainty to be low or very low for most comparisons, primarily due to risk of bias, imprecision, and inconsistency. The detailed ‘GRADE Summary of Findings’ is presented in Table 3.

**Table 3:** GRADE summary of findings for upper limb rehabilitation interventions.

Comparison	Outcome (FMA-UE improvement)	No. of participants (studies)	Effect (MD, 95% CI)	Certainty of evidence (GRADE)	Reasons for downgrading
**BCI-FES vs CT**	Motor function	233 (4 RCTs)	MD = 6.01 (2.19 to 9.83)	⬤◯◯◯ Low	Risk of bias, small samples
**BCI-FES vs FES**	Motor function	107 (4 RCTs)	MD = 3.85 (2.17 to 5.53)	⬤◯◯◯ Low	Lack of blinding, selective reporting
**BCI-FES vs tDCS**	Motor function	59 (2 RCTs)	MD = 6.53 (5.57 to 7.48)	⬤◯◯◯ Low	Very small sample size
**BCI-FES+tDCS vs BCI-FES**	Motor function	278 (5 RCTs)	MD = 3.25 (−1.05 to 7.55)	⬤◯◯◯ Very low	High heterogeneity, imprecision
**BCI-FES+tDCS vs tDCS**	Motor function	58 (2 RCTs)	MD = 6.05 (−2.72 to 14.82)	⬤◯◯◯ Very low	High risk of bias, inconsistency

Certainty of evidence was assessed using the GRADE framework. Certainty levels: ⬤⬤⬤⬤ High, ⬤⬤⬤◯ Moderate, ⬤⬤◯◯ Low, ⬤◯◯◯ Very low. FMA-UE, Fugl-Meyer Assessment of Upper Extremity.

### 3.3 Meta-analysis

#### 3.3.1 Network geometry

The network plot demonstrated BCI-FES as the most frequently investigated intervention (*n* = 7 studies), followed by BCI-FES-tDCS (*n* = 5). The most common direct comparisons were BCI-FES vs. BCI-FES-tDCS (5 studies) and BCI-FES vs. CT (4 studies), while BCI-FES vs. tDCS and BCI-FES-tDCS vs. tDCS had the fewest comparisons (2 studies each). The network plot is presented in Figure 1.

#### 3.3.2 Direct pairwise meta-analysis

Four studies (*n* = 363) directly compared BCI-FES and CT, showing substantial heterogeneity (I^2^ = 53.7%). The random-effects model yielded a pooled MD of 6.01 (95% CI: 2.19–9.83) (Figure 4). Given that the minimal clinically important difference (MCID) for FMA-UE is estimated at approximately 5–7 points, this effect size is at the threshold of clinical relevance, suggesting that the observed improvement may be meaningful for patients.

Four studies (*n* = 107) comparing BCI-FES and FES demonstrated no heterogeneity (I^2^ = 0%). The fixed-effect model produced an MD of 3.85 (95% CI: 2.17–5.53) (Figure 5).

Two studies (*n* = 59) evaluating BCI-FES versus tDCS showed no heterogeneity (I^2^ = 0%), with a fixed-effect MD of 6.53 (95% CI: 5.57–7.48) (Figure 6).

Five studies (*n* = 278) comparing BCI-FES-tDCS and BCI-FES exhibited substantial heterogeneity (I^2^ = 91.0%), resulting in a random-effects MD of 3.25 (95% CI: −1.05-7.55) (Figure 7). Such extreme heterogeneity indicates that pooling across trials may yield unstable or potentially misleading effect estimates.

Two studies (*n* = 58) assessing BCI-FES-tDCS versus tDCS showed significant heterogeneity (I^2^ = 72.2%), with a random-effects MD of 6.05 (95% CI: −2.72-14.82) (Figure 8).

#### 3.3.3 Network meta-analysis

The inconsistency model was close to statistical significance (*p* = 0.060). Although a consistency model was adopted, this borderline result suggests potential instability in indirect comparisons, which should be considered when interpreting the network estimates. SUCRA rankings for FMA improvement were: BCI-FES-tDCS (98.9), BCI-FES (73.4), tDCS (33.3), FES (32.4), and CT (12.0) (Figure 2). Funnel plot symmetry suggested minimal publication bias (Figure 3). However, with as few as 2–4 studies contributing to several comparisons, the ability to detect small-study effects was extremely limited. Moreover, some included trials reported implausibly large treatment effects within very short intervention periods, raising concern about potential reporting bias.

League table comparisons revealed BCI-FES and BCI-FES-tDCS were significantly superior to CT (*p* < 0.05), but no statistically significant differences were observed versus FES or tDCS (Table 4).

**Table 4:** League table of different intervention comparisons.

BCI-FES				
−2.53 (−7.60, 2.12)	BCI-FES + tDCS			
6.59 (0.92, 12.66)	9.13 (1.96, 17.16)	CT		
4.05 (−1.83, 9.82)	6.57 (−0.91, 14.43)	−2.59 (−11.02, 5.40)	FES	
1.45 (−5.84, 9.18)	4.02 (−3.17, 11.89)	−5.11 (−14.73, 4.18)	−2.58 (−11.95, 7.12)	tDCS

## 4 Discussion

This network meta-analysis compared the efficacy of BCI-FES, tDCS, and their combination in post-stroke upper limb rehabilitation. The combined BCI-FES and tDCS approach (SUCRA = 98.9) showed clear superiority over single interventions, yielding a 9.13-point improvement in FMA-UE compared with conventional therapy (95% CI: 1.96–17.16). These results provide important evidence for optimizing rehabilitation strategies. However, the findings mainly address impairment-level recovery (FMA-UE). Evidence for activity-level outcomes (ARAT, WMFT, ADL) was limited, and further studies are needed before assuming benefits in real-world function.

BCI-FES has shown considerable efficacy in promoting upper limb recovery after stroke, an effect likely rooted in its unique capacity for bidirectional central-peripheral neural remodeling. The BCI component operates by decoding sensorimotor rhythms—such as the *μ*-rhythm (8–12 Hz) or event-related desynchronization/synchronization (ERD/ERS) patterns—to enhance the encoding of motor intention within the primary motor cortex (15). This process delivers intention-contingent feedback, engaging motor planning and execution circuits to drive neuroplastic adaptation. In parallel, FES applies patterned electrical stimulation to activate paralyzed muscles, restoring efferent motor pathways. By integrating these technologies, BCI-FES establishes a closed-loop system that directly couples motor intention with muscle activation, strengthening neural transmission and improving muscular coordination to accelerate functional recovery (16). Neuroimaging evidence further indicates that BCI-FES training boosts functional connectivity within the ipsilesional M1 area by 32%, a change that correlates with FMA-UE gains and highlights its capacity to remodel motor networks (17). Consistent with these mechanisms, a multicenter randomized trial reported a 41% greater rate of independent finger movement recovery with BCI-FES compared to conventional therapy (OR = 3.2, 95% CI: 1.6–6.4) (18). Nevertheless, clinicians should interpret such benefits with discernment: improvements observed at the impairment level (e.g., FMA-UE) do not necessarily translate into meaningful gains in activity-based outcomes such as ARAT, WMFT, or activities of daily living.

The integration of BCI-FES with tDCS produces synergistic therapeutic benefits that surpass those achieved by either intervention alone. By modulating cortical excitability non-invasively, tDCS complements the closed-loop neuromodulation of BCI-FES, creating a multi-level rehabilitation strategy. Anodal tDCS enhances neuronal excitability and neurotransmitter dynamics, thereby priming the motor cortex for plasticity (19). Our network meta-analysis revealed that the combined approach achieved the highest SUCRA ranking, suggesting tDCS may boost BCI-FES decoding efficiency by approximately 18% through elevated cortical excitability thresholds (20). This potentiation may involve tDCS-driven modulation of NMDA receptor-dependent LTP, reinforcing synaptic efficacy in motor pathways (21). Additionally, the combined protocol appears to enhance contralesional corticospinal tract engagement, amplifying motor output to the affected limb—a finding consistent with Koyama et al.’s report of increased cross-hemispheric involvement following combined therapy (8).

This study indicates that BCI-FES provides superior precision compared to conventional FES by delivering stimulation synchronized with the patient’s motor intention, thereby promoting active engagement and targeted neural retraining. Unlike traditional FES—which effectively activates muscles but operates without real-time cortical feedback—BCI-FES establishes a closed-loop interaction that may more effectively induce neuroplasticity (14). Meta-analysis revealed that BCI-FES yielded significantly greater improvement in FMA-UE scores than FES alone (MD = 3.85, 95% CI: 2.17–5.53). However, this advantage did not reach the minimal clinically important difference (MCID ≈ 5–7 points), suggesting that while statistically significant, its clinical relevance may be modest. In contrast, the 6-point gain of BCI-FES over conventional therapy approaches the MCID threshold, indicating stronger clinical meaningfulness. Moreover, BCI-FES demonstrated clearer benefits over tDCS alone, likely owing to its dual mechanism: modulating central cortical excitability while simultaneously facilitating peripheral motor output via electrical stimulation (7, 22, 23). This integrated action supports more comprehensive neural recovery. Ongoing technological refinements in BCI-FES are expected to overcome efficacy plateaus, particularly in patients with severe paralysis, while improved usability and cost-effectiveness may expedite its integration into clinical practice. Future studies should explore hybrid strategies, such as BCI-FES combined with virtual reality or robotic assistance, to maximize functional outcomes and foster long-term recovery.

Despite the strengths of network meta-analysis, several limitations must be acknowledged. First, the methodological quality of included RCTs was mostly low to moderate, with frequent weaknesses in randomization, allocation concealment, and blinding. Many trials were small, single-center studies lacking pre-registration. These issues increase the risk of bias and reduce confidence in pooled estimates. Although RoB 2.0 was applied, its results were not fully incorporated into the interpretation. A formal GRADE assessment would likely downgrade most comparisons to low or very low certainty due to small samples, methodological flaws, and high heterogeneity. Thus, the conclusions should be interpreted with caution. Second, substantial heterogeneity across several comparisons (e.g., I^2^ > 90% in BCI-FES + tDCS vs. BCI-FES) poses a major concern, as pooling such heterogeneous trials risks generating misleading estimates. In addition, the NMA inconsistency test approached significance (*p* = 0.06), suggesting potential instability in indirect comparisons. These issues should be regarded as serious limitations rather than minor caveats. Although random-effects models were employed, variations in patient characteristics, intervention parameters, and treatment durations further compromise result stability. Moreover, limited direct comparisons between certain interventions may reduce estimation precision. Mechanistically, most studies relied solely on FMA-UE as an outcome, lacking multimodal neurophysiological assessments. In addition, the technical intricacy and economic viability of implementing combined BCI-FES/tDCS regimens necessitate further appraisal across varied clinical contexts. Significant methodological heterogeneity, particularly in the reporting of critical intervention parameters (tDCS intensity, FES specifications, BCI decoding accuracy), likely underlies the substantial statistical heterogeneity observed and highlights an imperative for standardized reporting conventions. While funnel plot asymmetry was not evident, the low statistical power to detect small-study effects and the presence of outliers with improbably large, short-term gains raise concerns about potential publication bias. Moreover, the paucity and inconsistency of activity-level outcome measures fundamentally limit inferences to the impairment domain. Therefore, despite the promising ranking, the evidence for the combined intervention—based on only five small RCTs with methodological limitations—must be considered preliminary and strictly hypothesis-generating at this stage.

## 5 Conclusion

This network meta-analysis indicates that combining BCI-FES with tDCS represents a promising, multimodal strategy for enhancing upper-limb motor recovery after stroke, likely through synergistic promotion of neuroplasticity. However, the current evidence remains preliminary and hypothesis-generating, as the limited number of available RCTs, methodological shortcomings, and substantial heterogeneity preclude definitive conclusions. Prior to formulating clinical recommendations, future multicenter, high-quality randomized trials with larger sample sizes are imperative to validate these findings. Subsequent research should prioritize standardizing stimulation protocols, optimizing individualized parameters, and implementing long-term follow-ups to firmly establish clinical efficacy and facilitate the translation of brain-computer interface technology into neurorehabilitation practice.

## Data Availability

The raw data supporting the conclusions of this article will be made available by the authors, without undue reservation.
